# Fast Phase-Contrast Cine MRI for Assessing Intracranial Hemodynamics and Cerebrospinal Fluid Dynamics

**DOI:** 10.3390/diagnostics10040241

**Published:** 2020-04-21

**Authors:** Naoki Ohno, Tosiaki Miyati, Tomohiro Noda, Noam Alperin, Takashi Hamaguchi, Masako Ohno, Tatsuhiko Matsushita, Mitsuhito Mase, Toshifumi Gabata, Satoshi Kobayashi

**Affiliations:** 1 Faculty of Health Sciences, Institute of Medical, Pharmaceutical and Health Sciences, Kanazawa University, 5-11-80 Kodatsuno, Kanazawa 9200942, Ishikawa, Japan; nohno@med.kanazawa-u.ac.jp (N.O.); satoshik@staff.kanazawa-u.ac.jp (S.K.); 2 Department of Radiology, Kobe University Hospital, 7-5-2 Kusunoki-cho, Chuo-ku, Kobe 6500017, Hyogo, Japan; kobu_kuro6@hotmail.com; 3 Department of Radiology, University of Miami, 1150 NW 14th Street, Suite 713, Miami, FL 33136, USA; NAlperin@med.miami.edu; 4 Department of Radiological Technology, Kanazawa University Hospital, 13-1 Takara-machi, Kanazawa 9208641, Ishikawa, Japan; gucciiin@med.kanazawa-u.ac.jp (T.H.); takanaga_masako@yahoo.co.jp (M.O.); 5 Division of Clinical Radiology Service, Kyoto University Hospital, 54 Kawaharacho, Shogoin, Sakyo-ku, Kyoto 6068507, Japan; tmmt_company@yahoo.co.jp; 6 Department of Neurosurgery, Nagoya City University Graduate School of Medical Sciences, 1-Kawasumi, Mizuho-cho, Mizuho-ku, Nagoya 4678601, Aichi, Japan; mitmase@med.nagoya-cu.ac.jp; 7 Department of Radiology, Kanazawa University Hospital, 13-1 Takara-machi, Kanazawa 9208641, Ishikawa, Japan; gabata@med.kanazawa-u.ac.jp

**Keywords:** phase-contrast, intracranial hemodynamics, cerebrospinal fluid hydrodynamics, respiration

## Abstract

We propose fast phase-contrast cine magnetic resonance imaging (PC-cine MRI) to allow breath-hold acquisition, and we compared intracranial hemo- and hydrodynamic parameters obtained during breath holding between full inspiration and end expiration. On a 3.0 T MRI, using electrocardiogram (ECG)-synchronized fast PC-cine MRI with parallel imaging, rectangular field of view, and segmented k-space, we obtained velocity-mapped phase images at the mid-C2 level with different velocity encoding for transcranial blood flow and cerebrospinal-fluid (CSF) flow. Next, we calculated the peak-to-peak amplitudes of cerebral blood flow (ΔCBF), cerebral venous outflow, intracranial volume change, CSF pressure gradient (ΔPG), and intracranial compliance index. These parameters were compared between the proposed and conventional methods. Moreover, we compared these parameters between different utilized breath-hold maneuvers (inspiration, expiration, and free breathing). All parameters derived from the fast PC method agreed with those from the conventional method. The ΔPG was significantly higher during full inspiration breath holding than at the end of expiration and during free breathing. The proposed fast PC-cine MRI reduced scan time (within 30 s) with good agreement with conventional methods. The use of this method also makes it possible to assess the effects of respiration on intracranial hemo- and hydrodynamics.

## 1. Introduction

The dimensions of the cranial cavity that houses the brain are quite constrained. Changes in intracranial hemodynamics and hydrodynamics due to intracranial lesions, including brain edema and hematoma, are tightly linked to both brain homeostasis and damage. Therefore, it is crucial to precisely monitor intracranial hemo- and hydrodynamic properties, namely, cerebral blood flow (CBF), cerebral venous outflow (CVO), cerebrospinal fluid (CSF) flow, intracranial pressure (ICP), and compliance [1]. Although invasive tests that require the insertion of a catheter with a pressure sensor into the cranium are commonly used for monitoring ICP and compliance [2,3], recent studies demonstrated the utility of magnetic resonance imaging (MRI) as an alternative, noninvasive diagnostic method [4,5,6,7,8,9]. Specifically, phase-contrast cine MRI (PC-cine MRI) has been widely used for the noninvasive measurement of blood and CSF flows and tissue motion. Miyati et al. proposed a noninvasive technique using electrocardiogram (ECG)-synchronized PC-cine MRI to determine intracranial hemo- and hydrodynamic parameters, including the intracranial compliance index (ICCI) and peak-to-peak amplitudes of intracranial volume change (ΔICVC) and the CSF pressure gradient (ΔPG) during the cardiac cycle [5]. They demonstrated that both ICCI and ΔICVC were significantly lower in patients with idiopathic normal pressure hydrocephalus than those in the control. Alperin et al. also used a similar technique and reported that patients with Chiari malformation had significantly lower ICCI and ΔICVC compared with control subjects [4]. However, conventional PC-cine MRI is relatively time-consuming (e.g., the total scan time for PC acquisition with two different velocity encodings that are required for ICCI analysis is over 5 min) and is therefore susceptible to patient movements, heart rate (HR) variability, and arrhythmia during the scan. Thus, faster PC acquisitions are desirable to minimize these effects and alleviate the burden on patients.

Intracranial hemodynamics and hydrodynamics are confounded by respiration [10]. Although Yildiz et al. concluded that respiratory effects can be averaged out over relatively long scan time for conventional PC-cine MRI [11], a study by Spijkerman et al. found that CSF flow was considerably confounded by respiration via PC–MRI with respiratory gating [12]. A solution to this respiratory confounder is a breath-hold scan. However, a change in the breath-hold maneuver, for example, at full inspiration or end expiration, may potentially affect intracranial hemodynamics and hydrodynamics.

The main purposes of this study were to (1) propose a fast PC-cine MRI to allow breath-hold acquisition, and (2) assess the validity of this method by comparing the ICCI, ΔICVC, ΔPG, and peak-to-peak amplitudes of CBF (ΔCBF) and CVO (ΔCVO) during the cardiac cycle with a conventional method. Moreover, we hypothesized that the measured intracranial hemo- and hydrodynamic parameters would be affected by differences in the utilized breath-hold maneuver. Therefore, we also compared these parameters during breath holding at both full inspiration and end expiration.

## 2. Materials and Methods

### 2.1. Overview of Proposed Fast Phase-Contrast Cine Magnetic Resonance Imaging (PC-cine MRI)

Acceleration of proposed fast PC-cine MRI was achieved by combining the parallel-imaging, rectangular-field-of-view (FOV), and segmented k-space techniques.

Parallel imaging with the array spatial-sensitivity-encoding technique (ASSET) is based on the sensitivity-encoding method [13,14]. This technique enables faster acquisition by what is known as the acceleration or reduction factor by reducing the number of sampled phase-encoding lines, although at a cost of reduced signal-to-noise ratio (SNR). For example, with an acceleration factor of 2 in our proposed method, only half of the k-space was sampled. Consequently, scan time was shortened by a factor of two. However, consequent reduction in the sampled phase-encoding lines, i.e., reduced phase-encoding FOV, with the ASSET introduces aliasing or wraparound artifacts in the Fourier-transformed image. The ASSET reconstruction algorithm in conjunction with a phased-array receiving coil addresses this problem. In the ASSET, the spatial-sensitivity profile of each individual phased-array element is acquired to restore a full FOV image. In theory, the signal-intensity differences between each coil element at any given pixel in an image is related to the spatial location of that point. With the relationship between sensitivities of individual coil elements and the signal intensity of the aliased image, aliasing artifacts can be unfolded and removed in ASSET reconstruction.

Similarly to parallel imaging, the rectangular FOV technique can also shorten scan time by reducing phase-encoding FOV, i.e., reducing the number of sampled phase-encoding lines, albeit at the expense of reduced SNR. Because the maximal and minimal amplitudes of the phase-encoding gradient are maintained, and both the phase-encoding FOV and the number of phase-encoding lines are reduced, this technique invariably maintains pixel size in the phase-encoding direction and overall spatial resolution. Aside from parallel imaging, a reduced FOV image is reconstructed. Therefore, given the combination of the ASSET technique, a slightly rectangular FOV of 80%, with minimal aliasing artifacts, was used in the proposed method, which enabled 1.25-fold faster acquisition.

**Figure 1:**
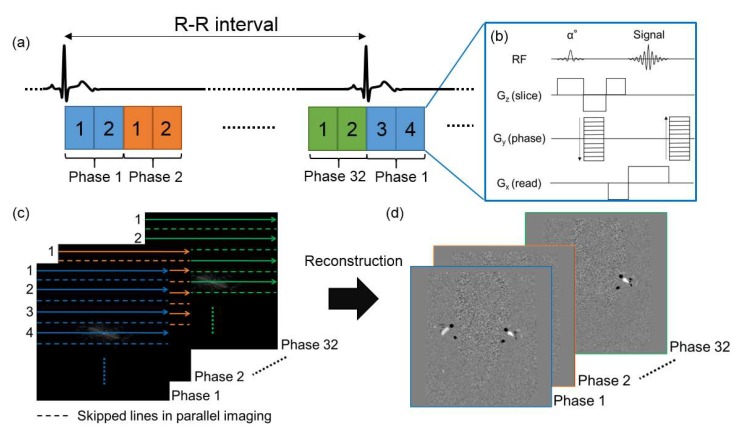
Schematic of fast electrocardiogram (ECG)-synchronized phase-contrast (PC) cine MRI with parallel-imaging and segmented k-space techniques. Each rectangle in (**a**) shows (**b**) PC gradient-echo sequence. (**c**) With a view per segment of 2, two phase-encoding lines are continuously acquired for each temporal phase, and filled in k-space with parallel-imaging acceleration factor of 2. (**d**) Velocity-mapped phase images are reconstructed.

In the segmented k-space technique, the R–R interval is divided into phases corresponding to k-space segments, and multiple lines of k-space are acquired during each cardiac cycle at the expense of reduced temporal resolution (Figure 1). The number of k-space lines acquired at each cardiac phase is referred to as views per segment (VPS). For example, in case of 2 VPS, two lines of k-space are consecutively acquired during a cardiac cycle and filled in the k-space. As a result, the number of cardiac cycles required to complete the k-space is reduced to half, allowing twofold faster acquisition. VPS is analogous to echo the train length with fast spin-echo imaging. Furthermore, this technique can reduce scan time without decreasing spatial resolution.

Consequently, a total of fivefold faster acceleration (2 × 1.25 × 2) was achieved in the proposed method by combining these three techniques.

### 2.2. Imaging Conditions

**Figure 2:**
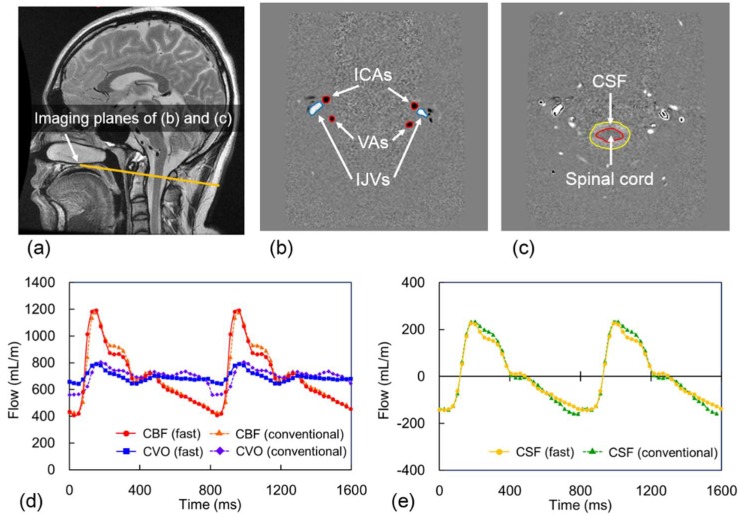
Examples of midsagittal T_2_-weighted and velocity-mapped PC images. (**a**) Imaging plane (yellow line) for ECG-synchronized PC-cine MRI. (**b**) Velocity-mapped PC-cine MRI of arterial inflow (right and left internal carotid arteries (ICAs) and vertebral arteries (VAs)) and venous outflow (both internal jugular veins (IJVs)). (**c**) Velocity-mapped PC-cine MRI of the cerebrospinal fluid (CSF) flow and spinal-cord displacement. Examples of volumetric flow waveform of (**d**) cerebral blood flow (CBF, sum of both side ICAs and VAs) and cerebral venous outflow (CVO, sum of both side IJVs and secondary cervical veins), and (**e**) CSF flow with proposed fast and conventional PC-cine MRI.

ECG-synchronized PC-cine MRI was performed in a 3.0 T MRI (Signa HDxt, GE Healthcare, Milwaukee, WI, USA) with a 12-channel phased-array head–neck coil to measure net transcranial flow (CBF, CVO, and CSF flow) and the displacement of the spinal cord during the cardiac cycle. Transverse-imaging planes were set at the mid-C2 level perpendicular to the internal carotid and vertebral arteries for blood flow, and to the spinal canal for CSF flow (Figure 2a) [5]. The velocity-encoding bipolar gradients were applied perpendicular to the imaging slices and set at 90 cm/s for blood flow and 7 cm/s for CSF flow and cord displacement. For the conventional PC method, a gradient-echo sequence was used with the following parameters: repetition time, 10 ms; echo time, 5.3 to 7.6 ms; flip angle, 15 degrees; FOV, 160 mm; voxel size, 0.62 × 1.0 × 5.0 mm^3^ (readout × phase × slice); number of averaged signals, 1; flow-compensation gradient; number of cardiac phases, 32; and approximate scan time for a HR of 70 beats per minute (bpm), 149 s. The proposed fast PC method was performed using identical imaging parameters as the conventional method except for an ASSET acceleration factor of 2, rectangular FOV of 80%, and VPS of 2. These modifications did not affect the spatial resolution. The approximate scan time of the proposed method for a HR of 70 bpm was 30 s, which is one-fifth of the conventional method.

### 2.3. Subjects

Twelve healthy subjects (ten men and two women, 22.6 ± 1.4 years of age) were scanned. The subject-inclusion criteria were an age of 20 to 30 years and no known history of neurological disorder or head trauma. This study was approved by the institutional review board. The purpose and procedures of our study were completely explained to all subjects, and scans were performed only after informed consent from each subject has been obtained. The proposed and conventional PC-cine MRI methods using free breathing for each subject were performed consecutively on the same day. We evaluated the relationships of ΔICVC, ΔPG, ICCI, ΔCBF, and ΔCVO values between the proposed and conventional methods using Spearman’s correlation coefficients and Bland–Altman plots to demonstrate the validity of the proposed method. The proposed PC-cine MRI was then performed during breath holding at full inspiration and at the end of expiration. We also compared the abovementioned parameters between different breath-hold maneuvers (inspiration, expiration, and free breathing) using the Wilcoxon signed-rank test with false discovery rate correction for multiple comparisons. All statistical analyses were performed in SPSS for Windows, version 25 (IBM, Chicago, IL, USA). A *p* value of < 0.05 was considered statistically significant.

### 2.4. Determination of Intracranial Hemo- and Hydrodynamic Parameters

Using the pulsatility-based segmentation method [15] incorporated into an analytical program (MR-ICP) developed by Alperin et al., we defined the lumen boundaries of the internal carotid and vertebral arteries (ICAs and VAs, respectively), internal jugular veins (IJVs), secondary cervical veins (e.g., vertebral, epidural, and deep cervical veins), and spinal canal on the velocity-mapped phase images (Figure 2b,c), and obtained the volumetric flow rates and displacement during the cardiac cycle within the defined lumens (Figure 2d,e). Then, the ICVC was calculated using the following equations [16].

*ICVC*(*t*) = [*V_a_*(*t*) − *V_v_*(*t*) − *V_c_*(*t*) − *V_s_*(*t*)] *Δt* (1)

Σ_Cardiac cycle_*ICVC*(*t*) = Σ[*V*_a_(*t*) − *V*_v_(*t*) − *V*_c_(*t*) − *V*_s_(*t*)] *Δt* = 0 (2)

where *V*_a_(*t*) is the arterial volumetric flow rate (the sum of both side ICAs and VAs), *V*_v_(*t*) is the venous volumetric flow rate (the sum of both side IJVs and secondary cervical veins), *V*_c_(*t*) is the CSF flow, and *V*_s_(*t*) is the spinal-cord displacement. Further, *t* is a time point during the cardiac cycle.

We determined the peak-to-peak ICVC during the cardiac cycle (ΔICVC) using the following equation [16].

*ΔICVC* = *ICVC*_max_ − *ICVC*_min_ (3)

where *ICVC*_max_ and *ICVC*_min_ are the maximal and minimal *ICVC* during the cardiac cycle, respectively.

Next, we calculated the craniospinal CSF PG during the cardiac cycle using the following simplified Navier–Stokes equation [16].

(4) $$PG=-\rho (\frac{\partial V}{\partial t}+V\cdot \nabla V)+µ\cdot \nabla^{2}V$$

where *ρ* is fluid density (1.0007 g/cm^3^), *μ* is fluid viscosity (1.1 cP), and *V* is the velocity vector. We assumed that CSF behaved as a Newtonian fluid. PG was calculated from 32 velocity-mapped phase images using a previously described method [16]: the inertial component of PG was estimated by first-order central difference, and the viscous component was derived from a pair of second-order central difference operators. Because PG decreases linearly with the CSF cross-sectional area due to pressure loss [16], PG was normalized by multiplying the CSF flow area to correct the effect. Then, ΔPG was calculated using the following equation [16].

*ΔPG* = *PG*_max_ − *PG*_min_ (5)

where *PG*_max_ and *PG*_min_ are the maximal and minimal *PG* during the cardiac cycle, respectively.

The ICCI was derived from the following equation [5].

*ICCI* = *ΔICVC*/*ΔPG* (6)

Finally, ΔCBF and ΔCVO were derived from the following equations [17].

*ΔCBF* = *CBF*_max_ − *CBF*_min_ (7)

*ΔCVO* = *CVO*_max_ − *CVO*_min_ (8)

where *CBF*_max_ and *CBF*_min_ are the maximal and minimal *CBF* during the cardiac cycle, respectively; and *CVO*_max_ and *CVO*_min_ are the maximal and minimal *CVO* during the cardiac cycle, respectively. CBF was calculated as the sum of the volumetric flow rates of both side ICAs and VAs, and CVO was calculated as the sum of the volumetric flow rates of both side IJVs and secondary cervical veins. Representative waveforms of CBF, CVO, ICVC, and PG during the cardiac cycle with the proposed fast and conventional methods are shown for a representative subject in Figure S1 of the Supplementary Material.

## 3. Results

The proposed fast PC method with parallel imaging, rectangular FOV, and segmented k-space acquisition reduced the scan time fivefold compared with the conventional method.

**Figure 3:**
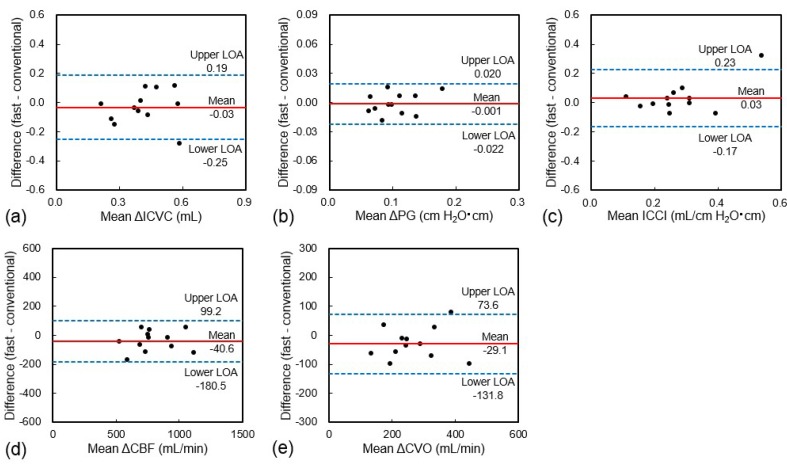
Bland–Altman plots showing differences in (**a**) ΔICVC, (**b**) ΔPG, (**c**) ICCI, (**d**) ΔCBF, and (**e**) ΔCVO during free breathing between proposed fast and conventional PC-cine MRI methods. Red solid line, mean difference; blue dashed lines, upper and lower limits of agreement (LOA, mean ± 2SD).

Table 1 and Figure 3 show the comparisons and Bland–Altman plots of ΔICVC, ΔPG, ICCI, ΔCBF, and ΔCVO during free breathing between the proposed fast and conventional PC-cine MRI methods, respectively. All derived parameters from the fast PC method agreed with those from the conventional method. On the other hand, the SNR of the fast PC method decreased by approximately 20% on average in comparison with the conventional method.

Table 2 shows ΔICVC, ΔPG, ICCI, ΔCBF, and ΔCVO during breath holding at full inspiration and at the end of expiration, and during free breathing using the fast PC method. The ΔPG during full inspiration breath holding was significantly higher than that at the end of expiration and during free breathing. The ΔCBF during full inspiration breath holding also tended to be higher; however, it did not reach significance compared with the value at the end of expiration and during free breathing. There were no significant differences in ΔICVC, ICCI, or ΔCVO between breath holding at full inspiration and at the end of expiration, and during free breathing.

## 4. Discussion

PC-MRI is a reliable flow quantitation method. A number of studies previously verified the accuracy and reliability of PC-MRI. Nevertheless, conventional PC-MRI is still slow (approximately 2 min and 30 s for a single velocity measurement) and therefore sensitive to patient movements, HR variability, and arrhythmia during the scan. Faster acquisition is desirable to make PC-based flow quantification more accurate and practical for clinical use. In the current study, we sought to accelerate PC-MRI by combining an existing 2D PC sequence with parallel imaging, rectangular FOV, and segmented k-space, and compared the intracranial hemo- and hydrodynamic parameters obtained with the proposed method with those obtained using the conventional method. The two methods showed good agreement with those parameters, suggesting comparable accuracy between the methods despite an approximately fivefold faster scan time with the proposed method. The correlation coefficient of ΔICVC was slightly lower than that of other parameters. ΔICVC was in the order of less than 0.1% of total intracranial volume, making reproducible and accurate measurements challenging. Previous studies demonstrated a large measurement variability for ΔICVC (more than 10%), and found that variability was mainly due to variations in the subject’s HR between the two separate PC-cine MRI scans for fast blood flow and slow CSF flow [1,18]. A relatively large difference in the waveform of ICVC during the cardiac cycle between the proposed fast and conventional methods may also be explained by lower reproducibility (see Figure S1c in Supplementary Material). The variability of ΔICVC can be improved using a dual velocity-encoding technique that allows the simultaneous acquisition of blood and CSF flow, minimizing the effect of the variation of a subject’s HR between both scans [1].

The accuracy of PC-MRI combined with parallel imaging was previously reported by Thunberg et al. [19], who found significantly increased noise and strong deviations of measured velocity in the central parts of the image at a parallel imaging acceleration factor of 3 or higher. Spatially varied noise, which depends on the geometry factor introduced in parallel imaging, leads to position dependency of accuracy on velocity measurements. This effect would be especially critical for flow measurements in ICAs, VAs, IJVs, secondary cervical veins, and the spinal canal, which are all located in the central parts of the image, that is, areas with a high geometry factor. Moreover, the combined use of parallel imaging and rectangular FOV in the proposed method further reduces the SNR. In fact, we found an SNR decrease of approximately 20% in the proposed method compared with the conventional method. Nevertheless, our results demonstrated that the intracranial hemo- and hydrodynamic parameters showed close correlation and agreement between both the methods. Therefore, the use of parallel imaging with an acceleration factor of 2 and rectangular FOV of 80% in the proposed method is considered acceptable and is less likely to affect flow-measurement accuracy.

Using the segmented k-space technique with two VPS, data acquisition could be completed in half the scan time at the expense of the small degradation of effective temporal resolution. Poutanen et al. noticed that increasing the number of VPS led to temporal smoothing of the velocity waveform during the cardiac cycle, thereby underestimating peak velocity at systole, and overestimating the lowest velocity at diastole [20]. Our results showed that the peak-to-peak changes in the hemo- and hydrodynamic parameters, which were expected to be strongly affected by the use of the segmented k-space technique, were generally comparable between the proposed and conventional methods. In other words, the proposed method using the k-space segmented technique with two VPS did not introduce a negative bias for the estimated velocity, and maintained the required temporal resolution for the determination of intracranial hemo- and hydrodynamic parameters. The proposed fast PC-cine MRI could be performed within approximately 30 s in the case of a 70 bpm HR, even without any specialized sequence, and would thus address the abovementioned issues.

Moreover, the proposed method is applicable to a single breath-hold PC-MRI, which allowed us to assess the effects of respiration on intracranial hemodynamics and hydrodynamics. Although flow measurements using breath-hold PC-MRI in cardiovascular diseases were reported by a number of groups [21,22,23], few studies evaluated intracranial hemo- and hydrodynamic parameters during breath holding [11,24]. Therefore, the increased understanding of changes in these properties in response to respiration may be gained from PC-MRI data during breath holding. This is the first study that performed breath-hold PC-MRI at full inspiration and at the end of expiration, and compared intracranial hemo- and hydrodynamic parameters between both breath-hold maneuvers. Our results demonstrated that ΔPG and ΔCBF during breath holding at full inspiration were higher than those at the end of expiration and free breathing, whereas there were no significant differences in ΔICVC, ICCI, or ΔCVO between the breath-hold maneuvers. In addition, ΔPG, ΔCBF, and ΔCVO during free breathing presented intermediate values between full inspiration and the end of expiration. These results indicated that intracranial hemo- and hydrodynamic parameters are confounded by respiration effects. The observed changes in ΔPG and ΔCBF during breath holding at full inspiration compared with at the end of expiration may be explained by changes in intrathoracic pressure over the respiratory cycle: Intrathoracic-pressure changes depend on the movement of the diaphragm during the respiratory cycle, that is, diaphragm contraction at the end of expiration decreases intrathoracic pressure, whereas diaphragm expansion at full inspiration increases pressure. An approximate change in intrathoracic pressure of 7 cmH_2_O between full inspiration and at the end of expiration was observed [25]. Inspiration decreases intrathoracic pressure, which increases venous return to the heart, and subsequently induces increased arterial pressure and inflow into the cranium [26], supporting our finding that ΔCBF increased at full inspiration compared with levels at the end of expiration. Increased arterial inflow into the cranium results in a small temporal increase in intracranial volume, which also causes a temporal increase in intracranial pressure [27]. At this time, both venous outflow and CSF flow from the cranium to the spinal canal occur immediately following increased arterial inflow to accommodate the increase in intracranial pressure [28]. As the intracranial-pressure waveform depends on the arterial-inflow waveform as the driving force [29], the increase in ΔPG reflects an increase of intracranial pressure produced by an increase of CBF after full inspiration. Our results suggested that the comparison of ΔPG with both breath-holding maneuvers at full inspiration and at the end of expiration can be used as a noninvasive stress test to assess intracranial hydrodynamics, which may enhance the sensitivity of the detection of impaired pressure-compensation capacity. Such respiratory stress would be implemented in clinical practice. Therefore, the method described here is diagnostically useful in patients with low pressure-compensation capacity, for example, idiopathic intracranial hypertension, Chiari malformation, or aqueductal stenosis.

The present study was based only on a limited number of healthy subjects, which makes it difficult to demonstrate the clinical usefulness of the proposed method. Further studies that include a larger number of healthy controls, and patients with impaired intracranial hemodynamics and hydrodynamics are needed to demonstrate the method’s validity. Moreover, relatively long breath holding for more than 30 s may be problematic in elderly patients. Thus, further acceleration, e.g., compressed sensing or real-time echo-planar imaging, should be undertaken to improve the clinical utility of the proposed method. Although there are the other fast PC techniques [30,31], these require special sequence and reconstruction. Therefore, we emphasize that the proposed method is considered to be of high clinical value because it does not require special sequence or reconstruction and can be reproduced by any MRI systems.

## 5. Conclusions

The proposed fast PC method with parallel imaging, rectangular FOV, and segmented k-space reduced scan time approximately fivefold with good agreement with the conventional method. This alternative method also allows breath-hold acquisition and the assessment of the effects of respiration on intracranial hemodynamics and hydrodynamics.

## Figures and Tables

**Table 1:** Comparison of proposed fast and conventional PC-cine MRI methods in terms of peak-to-peak intracranial volume change (ΔICVC), peak-to-peak pressure gradient (ΔPG), intracranial compliance index (ICCI), peak-to-peak cerebral blood flow (ΔCBF), and peak-to-peak cerebral venous outflow (ΔCVO).

Parameter	Fast	Conventional	R	*p* value
ΔICVC (mL)	0.40 ± 0.14	0.43 ± 0.13	0.748	0.005
ΔPG (cm H_2_O·cm)	0.104 ± 0.036	0.105 ± 0.032	0.937	<0.001
ICCI (mL/cm H_2_O·cm)	0.29 ± 0.15	0.26 ± 0.09	0.825	0.001
ΔCBF (mL/min)	775.7 ± 184.6	816.3 ± 176.5	0.867	<0.001
ΔCVO (mL/min)	254.4 ± 98.4	283.5 ± 90.6	0.846	0.001

Mean ± standard deviation, Spearman’s correlation coefficient (R), and *p* value shown for each variable; *p* value of < 0.05, statistically significant correlation between proposed fast and conventional methods.

**Table 2:** Comparisons of fast PC-cine MRI during breath holding at full inspiration (Ins) and at the end of expiration (Exp) and during free breathing (FB) in terms of peak-to-peak intracranial volume change (ΔICVC), peak-to-peak pressure gradient (ΔPG), intracranial compliance index (ICCI), peak-to-peak cerebral blood flow (ΔCBF), and peak-to-peak cerebral venous outflow (ΔCVO).

Parameter	Ins	Exp	FB	*p* Value		
				Ins vs. Exp	Ins vs. FB	Exp vs. FB
ΔICVC (mL)	0.45 ± 0.15	0.46 ± 0.19	0.44 ± 0.14	0.790	0.790	0.790
ΔPG (cm H_2_O·cm)	0.108 ± 0.035	0.086 ± 0.027	0.090 ± 0.019	0.015	0.018	0.530
ICCI (mL/cm H_2_O·cm)	0.31 ± 0.29	0.26 ± 0.26	0.25 ± 0.15	0.308	0.308	0.308
ΔCBF (mL/min)	778.2 ± 132.6	714.4 ± 90.0	749.5 ± 99.8	0.051	0.308	0.051
ΔCVO (mL/min)	297.5 ± 84.2	220.7 ± 71.4	255.5 ± 103.9	0.180	0.408	0.754

Mean ± standard deviation shown for each variable; *p* values calculated from Wilcoxon signed-rank test with false discovery rate correction for multiple comparisons; *p* value of <0.05, statistically significant difference between different breath-hold maneuvers.

## Supplementary Materials

Representative waveforms of CBF, CVO, ICVC, and PG during the cardiac cycle with the proposed fast and conventional methods for a representative subject are available online. Supplementary materials can be found at https://www.mdpi.com/2075-4418/10/4/241/s1.

## Abbreviations

CBF	cerebral blood flow
CVO	cerebral venous outflow
CSF	cerebrospinal fluid
ICP	intracranial pressure
PC-cine MRI	phase-contrast cine magnetic resonance imaging
ICCI	intracranial compliance index
ICVC	intracranial volume change
PG	CSF pressure gradient
ΔICVC	peak-to-peak amplitude of ICVC during the cardiac cycle
ΔPG	peak-to-peak amplitude of PG during cardiac cycle
HR	heart rate
∆CBF	peak-to-peak amplitude of CBF during the cardiac cycle
ΔCVO	peak-to-peak amplitude of CVO during the cardiac cycle
FOV	field of view
ASSET	array spatial sensitivity encoding technique
ICAs	internal carotid arteries
VAs	vertebral arteries
IJVs	internal jugular veins
SNR	signal-to-noise ratio
LOA	limit of agreement
Ins	inspiration
Exp	expiration
FB	free breathing
