# Supplementary material for: Fast Phase-Contrast Cine MRI for Assessing Intracranial Hemodynamics and Cerebrospinal Fluid Dynamics
Source: Diagnostics (Basel). 2020 Apr 21;10(4):241. doi: 10.3390/diagnostics10040241 (PMC7236008; doi:10.3390/diagnostics10040241)
Supplement: Supplementary file 1 [file diagnostics-10-00241-s001.pdf]

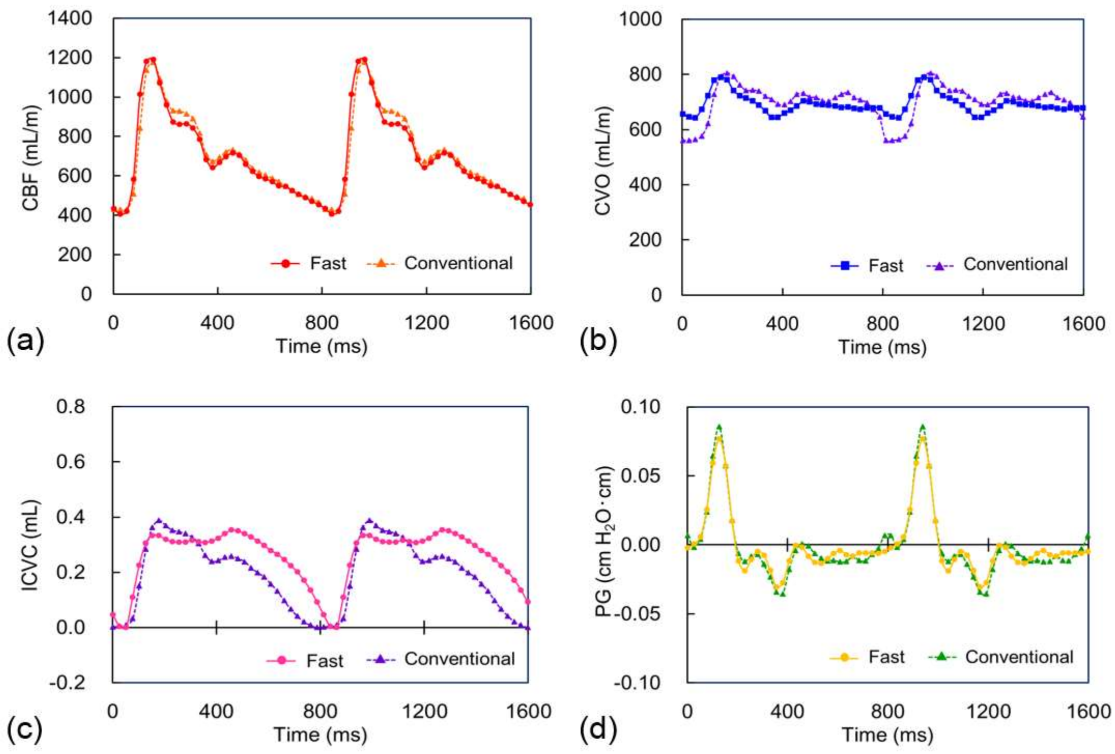

Figure S1. Representative waveforms of (**a**) cerebral blood flow (CBF), (**b**) cerebral venous outflow (CVO), (**c**) intracranial volume change (ICVC), and (**d**) pressure gradient (PG) during the cardiac cycle with the proposed fast and conventional methods are shown for a representative subject.
